# Cervical adenoid basal carcinoma associated with invasive squamous cell carcinoma: A report of rare co-existence and review of literature

**DOI:** 10.1186/1477-7819-9-132

**Published:** 2011-10-18

**Authors:** Boonlert Viriyapak, Sung Taek Park, Ah Won Lee, Jong Sup Park, Chung Won Lee, Min Jong Song, Soo Young Hur

**Affiliations:** 1 Department of Obstetrics and Gynecology, Faculty of Medicine Siriraj Hospital, Mahidol University, Bangkok, Thailand; 2 Department of Obstetrics and Gynecology, Seoul St. Mary's Hospital, The Catholic University of Korea, Seoul, Republic of Korea; 3 Department of Pathology, Seoul St. Mary's Hospital, The Catholic University of Korea, Seoul, Republic of Korea

**Keywords:** Adenoid Basal, Squamous Cell, Carcinoma, Cervix Uteri

## Abstract

Cervical adenoid basal carcinoma (ABC) rarely can harbor associated malignancies like adenoid cystic carcinoma or squamous cell carcinoma (SCC), which express markedly different prognosis from a pure ABC, making an appropriate biopsy essential to provide a clear diagnosis and therapeutic plan. We report a 64-year-old asymptomatic lady with an abnormal cervical cytology, who underwent a conization to reveal an ABC with overlying microinvasive SCC. Doubtful resection margins led us to perform radical hysterectomy with lymph node dissection. Subsequent pathological examination showed a true invasive SCC co-existing with ABC, with invasion of the parametrium. Unlike the indolent course of many pure ABC patients, the prognosis of 11 previously reported co-existing invasive SCC with ABC patients appears to depend on the SCC component. Our case reiterates the importance of adequate biopsy with careful interpretation to cover the possibility of a co-existent malignancy. Besides, it presents an argument in favor of radical surgery for the primary treatment of suspicious associated malignancy, and supports adjuvant treatment according to the unfavorable extent of the co-existent invasive carcinoma.

## Background

Adenoid basal carcinoma (ABC) is a rare cervical malignancy accounting for less than 1% of all cervical cancers [1]. This tumor closely resembles adenoid cystic carcinoma (ACC), and in fact has been reported as the same entity in some early reports [2], till the first description by Baggish and Woodruff [3] in 1966. The indolent clinical course and an excellent prognosis unique to typical or pure ABC are well recognized. Most patients are asymptomatic, without a detectable cervical mass and are successfully treated by a non-radical surgery with excellent results. In contrast, ACC and basaloid squamous cell carcinoma (BSCC), which morphologically closely resemble ABC, have an aggressive clinical course often associated with recurrence and metastasis [4-6]. This makes accurate distinction between ABC and its morphological counterparts fairly important.

The association of ABC with other malignancies has been inconstantly reported [4-13]. As the ABC rarely showed malignant behavior, the prognosis appears to depend on the associated malignancy component which also decides the treatment protocol [12]. Although the association of ABC with other malignancies has been reported from time to time, there is paucity of data regarding the specific clinical course, the appropriate diagnostic procedure and the recommended treatment approach.

We report an uncommon association of an invasive squamous cell carcinoma (SCC) with an ABC. The clinical and histological features with the specific immunostains for histogenetic studies are described. Simultaneously, literatures for all reports of these rare co-existent malignancies are reviewed.

## Case presentation

**Figure 1:**
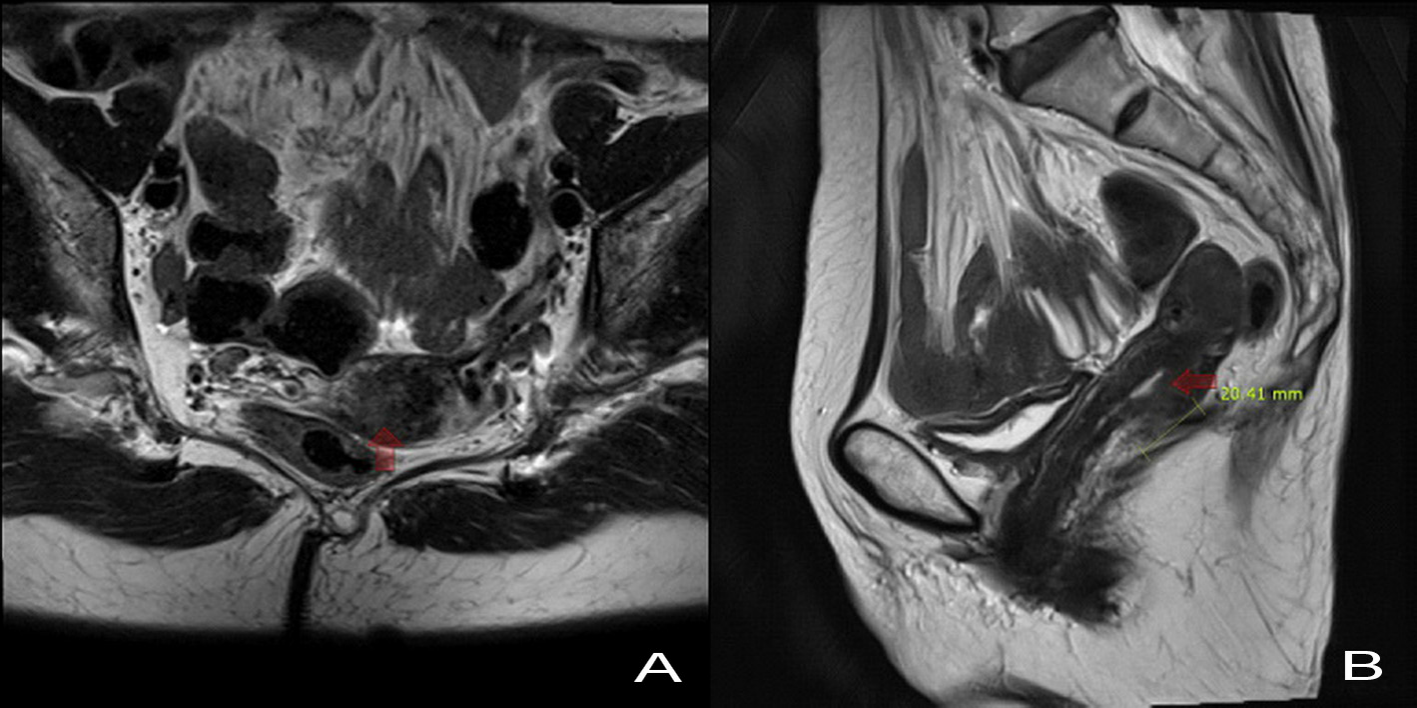
**T2-weighted pelvic magnetic resonance images on the axial plane (A) and sagittal plane (B)**. T2-weighted pelvic magnetic resonance images on the axial plane (A) and sagittal plane (B). Note the cervical mass-like lesion (infiltrative wall thickening with signal enhancement, 2.0 × 0.6 cm) at posterior portion of the uterine cervix with likely posterior vaginal fornix involvement. (arrows point to cervical lesion)

**Figure 2:**
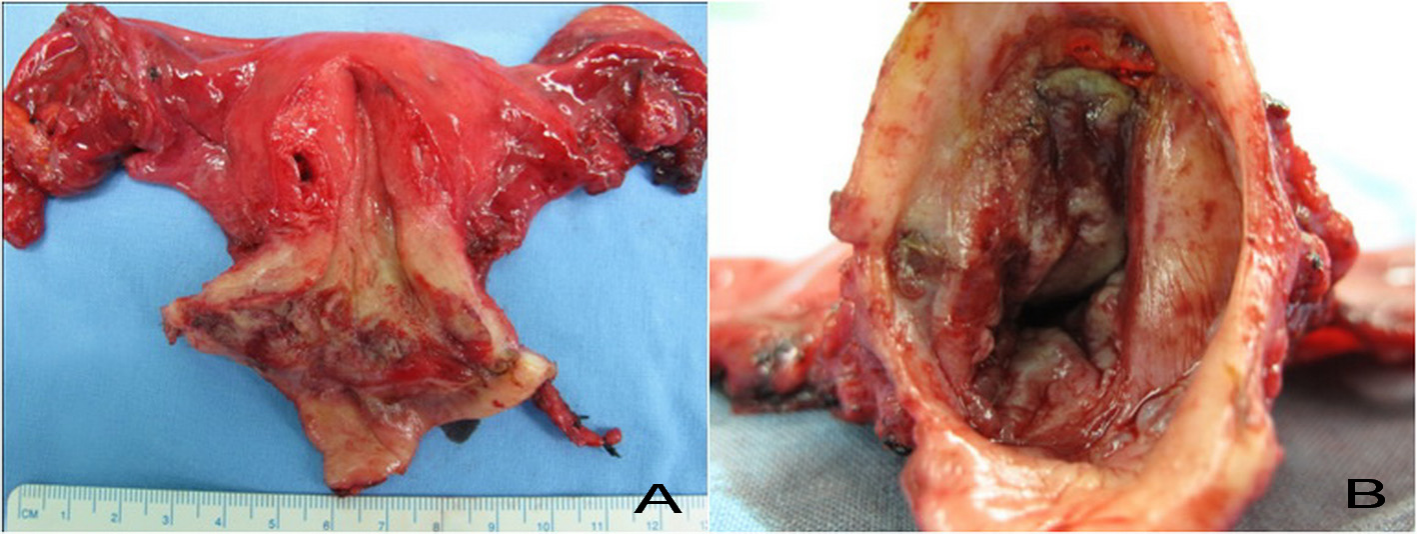
**Radical hysterectomy**. Radical hysterectomy. (A) Whole fresh gross specimen (cervix was opened at 12 o' clock position). (B) The cervix, showing ragged avulsion status post conization, no gross definite mass (x1.5 relatively magnified from Figure 2A).

A 64-year-old Korean female, presented with abnormal cervical cytology screening compatible with "squamous cell carcinoma", no visible cervical lesion was noted on the pelvic examination and the subsequent colposcopy. She has been menopause for 8 years ago and had no history of other gynecologic problem. She therefore underwent a cone biopsy of the cervix with loop electrosurgical excisional procedure (LEEP), which revealed ABC associated with microinvasive SCC with the tumors presented at endocervical margin. The remaining cervix was too small to do a repeat cone biopsy, so a pelvic magnetic resonance imaging (MRI) was proposed to find out an occult malignancy. MRI revealed an enhancing mass (2.0 × 0.6 cm) involving posterior lip of the cervix with a conclusion of cervical carcinoma (Figure 1). There is no evidence of distant metastasis or any suggestive metastatic lymph node by a positron emission tomography-computed tomography (PET-CT). Clinically stage IB was suggested and following radical hysterectomy, bilateral adnexectomy with pelvic and para-aortic lymph node dissection was undertaken uneventfully (Figure 2A). The pathologic diagnosis was adenoid basal carcinoma co-existing with invasive squamous cell carcinoma. The majority of tumor was ABC component (about 85%) merged with the minor areas of invasive SCC component (about 15%). There was no evidence of tumor in sections taken from 40 lymph nodes. The removed vagina and all resection margins were clear. Due to deeply infiltration into the stroma and invasion through the right parametrium mostly by the ABC component, post-operative cisplatin-containing chemoradiation was prescribed to limit the risk of recurrence. After that, she was clinically monitored and stated as no evidence of disease for up to 6 months interval.

**Figure 3:**
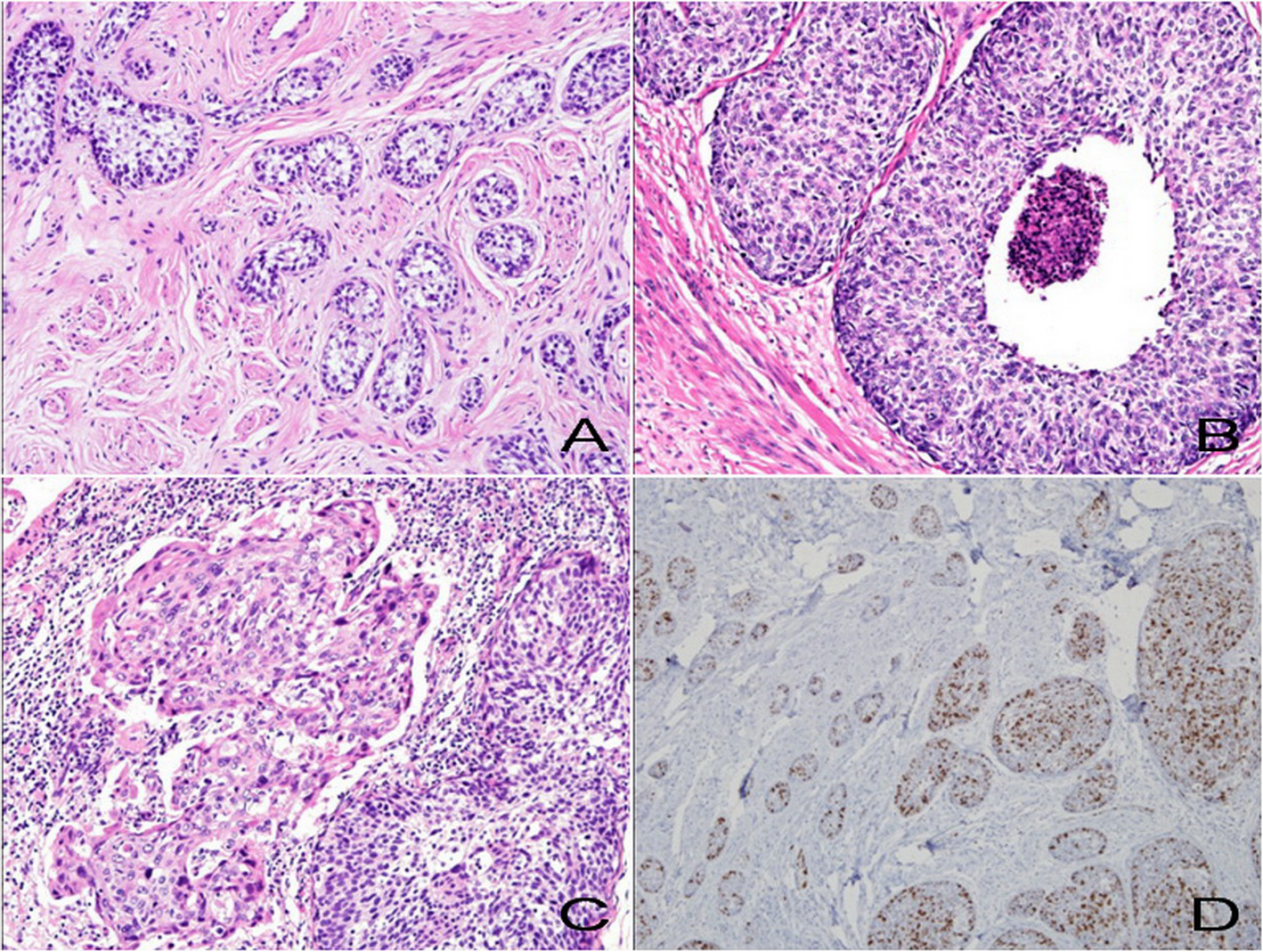
**Microscopic findings of the cervical co-existent tumors**. Microscopic findings of the cervical co-existent tumors. (A) The cervical stroma with infiltrated small basaloid nests of adenoid basal carcinoma component without stromal desmoplastic reaction (hematoxylin and eosin, ×200). (B) The admixed larger nests of squamous cell carcinoma component with central necrosis, and (C) severe nuclear atypia (hematoxylin and eosin, ×200). (D) The Ki-67 immunohistochemical stain, showing a higher index of staining in squamous cell carcinoma component than adenoid basal carcinoma component (×200).

On pathological examination, the LEEP specimen contained no gross definite lesion. Microscopically, there was diffuse infiltration of small basaloid nests and overlying cervical intraepithelial neoplasia (CIN) grade 3. The CIN lesion showed a focus of microinvasion, compatible with microinvasive SCC. The discrete nests composed of small and uniform basaloid cells with scanty cytoplasm and minimal nuclear atypia. The peripheral palisading morphologic pattern of the tumor nests was typically found and consistent with ABC lesion. The endocervical resection margin was extensively involved by tumor. The subsequent radical hysterectomy specimen showed ulceration due to previous conization without definite mass formation (Figure 2B). However, a transection of posterior cervix revealed a 2.1 × 1.8 × 0.9 cm growth infiltrated through the stroma. There was also diffuse infiltration of small basaloid nests without stromal desmoplastic reaction (Figure 3A). Merging with the multi focal areas of ABC component was the invasive SCC component, which is characterized with irregularly shaped larger nests and occasional central necrosis (Figure 3B). Nuclear atypia and high mitotic figures were noted (Figure 3C). Both components showed up to 0.9 cm stromal invasion within the total 1.2 cm stromal depth while focally involvement of right parametrium mainly by ABC was observed. The immunohistochemical stains were parallelly performed. A stain for p63 and Bcl-2 showed diffuse positive in both ABC and SCC components (data not shown). While a higher index of Ki-67 staining was observed with squamous cell carcinoma component (40%) than adenoid basal cell carcinoma component (25%) (Figure 3D).

## Conclusions

Among different malignancies in the classification of basaloid lesions of the uterine cervix proposed by Grayson and Cooper [14], there was a spectrum of basaloid carcinomas which were stated of histopathologic similarities. Many literatures of ABC have focused on diagnostic distinction from other mimicked aggressive malignancies such as ACC, BSCC and small cell (neuroendocrine) carcinoma and to elucidate its histopathogenesis. Meanwhile some reports also revealed valid evidences of the associated malignancy within morphologically pure ABC [4-17]. Since this association was initially documented in 1971 [7], most of the associated malignancies have been reported mixedly with pure ABCs in the early literatures. The concept of co-existence was then clarified by the divergent epithelial differentiation of the ABC lesion producing associated squamous, adenosquamous or rarely adenomatous malignant components which support the histogenetic pluripotent reserve cell origin of ABC [9]. The co-existing ABC with occasionally identified transitional areas to other malignancy mainly ACC, also showing the potential precursor concept [5,9]. Additionally, an associated malignancy could occur by the co-incidence of transformed tumor by the common pathogenetic high-risk human papillomavirus (HPV) integration in both components [10-12]. Although the precise frequency of these rare co-existences is hampered by the selection bias on the case-report evidences, it has shown a substantial account [18] and thus stresses the significance of careful approach with a suspect of associating malignancy in any provisional diagnosed ABC.

Recently, including our case, there are total 98 reported morphologically diagnosed ABCs [1-19]. Apart from the pure lesions, there are 27 cases (27.5%) described by ABC associated with other invasive malignancy in a single neoplasm. Regarding to this review, we firstly determine the common clinicopathological features of these specified cervical co-existent tumors which were sequentially summarized in the Additional File 1 table 1. Most of the patients were menopausal non-Caucasian women. The mean age was 66.7 years (44-84). The most frequent co-existing histology was SCC which accounts for 12 cases, ACC as second most found was co-existed in 9 cases while only 2 associated adenocarcinomas had been specified.

Typically, pure ABCs are asymptomatic, frequently presented by abnormal Pap smear which often associated with high grade squamous intraepithelial lesions (> 90%) [5,18]. These patients have been successfully treated by non-radical surgery such as simple hysterectomy without documented recurrence or tumor-related death. While if associated with other malignancy, 11 (52.6%) in 19 assessable presentations were asymptomatic with abnormal cervical cytology and most of these had no gross cervical lesion, implying that an associated invasive carcinoma is possibly presented by inactive clinical features. Two patients with co-existent malignancy including our case reported malignant cytology which less likely occurred in the pure ABC group. Moreover, even on comprehensive colposcopy, unusual ulcerative lesion of ABC or other infiltrative malignancy may not be detected. Thus, a cone biopsy is recommended to cover the most represented site of entire cervical tumors [20]. It was recorded that this procedure yielded a high definite diagnosis rate for the co-existent malignancy especially for these asymptomatic undetectable mass patients (6 in 9 patients, 66.6%). Comparing this data, the reviewed co-existent cases with a cervical mass at the presenting time showed its poorer outcome. It would be explained by undiagnosed associated SCC component resulted from an only cervical biopsy and inadequate treatment course for the real status of the associated invasive carcinoma.

In the LEEP specimen, only a focus of microinvasive SCC overlying multiple small nests of ABC was diagnosed. Suggesting that theoretically, area of true invasive carcinoma may not represent in all tumor area or generally admixed with ABC component, but can produce a distinct infiltrate through the deep part of stroma [1,12] and that resulted in possibility of inadequate assessment by an excisional biopsy. The endocervical resection margin which markedly positive mostly by ABC indicated that we could not exclude a hiding admixed ACC or a further invasive SCC accompanied in the remaining cervix and a repeat conization would be recommended [21]. Considering small residual cervix in the after-conization menopausal patient, a re-conization may be at risk of accidental injury to bladder or rectum with remarkable bleeding or subsequent infection [20]. High accuracy imaging to detect cervical tumor or local invasion by MRI (77-90% accuracy) [22] was a reasonable option and was again proved by our case. Also with concern on a risk of para-aortic lymph node spreading at a rate of 2-4% for this clinical stage IB1 cervical cancer [23], we additionally performed para-aortic lymph node dissection at the time of radical hysterectomy, bilateral salpingooophorectomy and pelvic lymph node dissection. The final revealed co-existence compiling ABC associated with truly invasive SCC supported our idea to treat this initially indefinite invasive diagnosed patient as a patient with locally invasive malignancy instead of doing simple hysterectomy which is described as the recommended operation for a typical ABC or even with microinvasive SCC.

The pathological diagnosis was obtained by a matching with the described morphologic criteria based on the current World Health Organization histological classification of tumors of the uterine cervix [1]. The infiltrating foci of the ABC component showed typical small uniform basaloid cell nest without desmoplastic stromal reaction. Squamous differentiation was diffuse and more frequently detected at the superficial area and focal area adjacent to the SCC component, forming cellular transition to the associated carcinoma [4,9]. These histomorphological findings were clearly distinct the ABC component from the other tumors with basaloid feature like ACC and BSCC which commonly presented with high nuclear pleomorphism, mitotic figure and cribriform or solid growth pattern [14]. Occupying at the most central part of tumors was the invasive squamous component which was deeply infiltrating tumor composed of larger nests of pleomorphic large, non-keratinized squamous differentiated cells with severe nuclear atypia and frequent mitotic features. Obviously this tumor presented a high growth appearance and highly invasive pattern by showing central necrosis and extensive stromal desmoplastic reaction respectively [1]. The result of p63 stain was typical in our case [17] which showed diffuse expression in both ABC and SCC components intensively by the palisading basaloid cells and suprabasal squamous differentiated cells in the central area of the superficial nests, while markedly reduced reaction in focal glandular (adenoid) differentiated area. The selective Bcl-2 positive reaction in basaloid tumor and SCC shown in our sections helpfully confirmed the basaloid squamous histogenesis and supported their neoplastic natures by showing anti-apoptotic genetic alteration in both components [18]. Moreover, for the cell cycle activity correlated Ki-67 stain which generally reduced expression in the basaloid and adenoid area [16,17], it was similar to our low index for ABC component and different to a well-defined stronger Ki-67 expression by SCC component. This was implied to higher proliferative activity and indicated distinct malignant potential of the invasive SCC, which corresponded to the central necrosis finding observed on the morphologic exam.

Likewise report of pure ABCs, almost all patients in co-existence group were clinically stage I which primarily treated by a type of hysterectomy. Along with 19 sufficient treatment data, the specific treatments were prescribed mostly depend on nature and stage of the revealed associated invasive malignancy either before or after primary surgery. Obviously different from the pure ABCs, only 3 co-existent cases (15.7%) have been treated by exclusively non-radical hysterectomy. Postoperative adjuvant therapies mainly by radiation were given to 5 cases (26.3%) with 2 of these had received adjuvant treatment after inadequate simple hysterectomy. This data would suggest that a primary radical treatment according to the extension of associated malignancy formally by radical hysterectomy with lymphadenectomy [23] should be recommended and may prevent unnecessary exposures to radiotherapy and/or chemotherapy.

Including our case with 6 months disease-free interval after postoperative chemoradiaton, the reviewed treatment outcomes of the ABC with associated malignancy patients had mean follow-up time of 31.1 months (0-120) with no evidence of disease in overall 13 of 20 retrievable cases (65.0%). Unlike the favorable outcome of pure ABCs, recurrences were found in 4 cases (20.0%) and 3 of these died of disease.

An invasive malignancy may simultaneously associated with any cervical ABC, that need an adequate diagnostic cone biopsy which can provide clear diagnosis and would serves both needs of proper radical therapy if a likely ABC with co-existent malignancy is diagnosed and also to prevent the over treatment of a pure ABC. Occasionally, if indefinite tissue diagnosis, an acceptable diagnostic option like MRI may be in preference. Through a decision with suspicious occult invasive carcinoma, the primary radical surgery should be considered. In advance, proper adjuvant treatment such as post-operative radiotherapy would be suggested according to the extent of the associated invasive malignancy.

## Consent

Written informed consent was obtained from the patient for publication of this case report and any accompanying images. A copy of the written consent is available for review by the Editor-in-Chief of this journal.

## Competing interests

The authors declare that they have no competing interests.

## Authors' contributions

BV contributed mainly in designing, literature review and writing work. SYH, the corresponding author who provided the case, planed and approved the written work. STP helped in correction of the manuscript. AWL performed the histopathologic process and described all findings and JSP gave advices and edited the discussion. CWL and MJS worked on the clinical presentation. All authors read and approved the final manuscript.

## Supplementary Material

Additional file 1 **Table 1**. Literature summary of reported cases of diagnosed adenoid basal carcinoma with associated invasive malignancy of the uterine cervix. Click here for file
