# Supplementary material for: Cervical adenoid basal carcinoma associated with invasive squamous cell carcinoma: A report of rare co-existence and review of literature
Source: World J Surg Oncol. 2011 Oct 18;9:132. doi: 10.1186/1477-7819-9-132 (PMC3217854; doi:10.1186/1477-7819-9-132)
Supplement: Supplementary file 1 [file 1477-7819-9-132-S1.DOC]

**Table 1. Literature summary of reported cases of diagnosed adenoid basal carcinoma with associated invasive malignancy of the uterine cervix†**

| No. | Authors[reference] / No. of patients | Age(yrs) | Race | Presentation | Pap smear findings | Gross cervical lesion | Associated CIN | Associated invasive malignancies | Definite diagnostic specimen | Stage‡ (clinical) | Therapy | Lymph node status | Distant metastasis | Follow-up (status,months) |
|---|---|---|---|---|---|---|---|---|---|---|---|---|---|---|
| 1 | 1971 Baggish & Woodruff [7] / 2 | 70 | Africans | PMB | class III | NI | none | Cylindroma | cervical biopsy on D&C | I | died before therapy | NA | none | DOC, after D&C |
| | | 69 | Africans | cervical mass | NA | cervical mass | none | Epidermoid carcinoma,Cylindroma | autopsy sections | IIB(initial) | RT | NA | lung(recurrent stage) | DOD, 10 yrs |
| 3 | 1980 Daroca & Dhurandhar [6] / 2 | 44 | Africans | vaginal bleeding | class V | cervical erosion | none | Infiltrating carcinoma | RH (after cervical biopsy) | I | RH, BSO, LND | negative | none | NED, 93 |
| | | 71 | NI | asymptomatic, abnormal Pap smear | class IV | NI | none | ASC | RH (after cold knife conization) | IB | RH, BSO, LND | negative | none | DOC, 3 |
| 5 | 1988 Ferry & Scully [5] / 1§ | 72 (mean) | NI | NI | NI | cervical mass | CIS | combined ABC-ACC‖, SCC | NI | NI | NI | NI | NI | NI |
| 6 | 1998 Clement et al [8] / 2 | 70 | NI | cervical mass | abnormal smear, not specified | cervical mass | CIS | SCC, CS (BSCC, ACC, SCC as carcinomatous component) | TH | IB | TH, BSO, pelvic LND | negative | none | pelvic recurrence, DOD, 3.5 yrs |
| | | 78 | NI | mass in vagina | NA | mass prolapsing into vagina | CIS | SCC, CS (SCC as carcinomatous component) | TH | IB | TH, BSO, para-aortic LND, RT | negative | none | NED, 21 |
| 8 | 1998 Brainard & Hart [4] / 1¶ | 67 | NI | asymptomatic, abnormal Pap smear | HSIL | none | none | SCC | cold-knife conization | IA2 | RH, LND | negative | none | NED, 28 |
| 9 | 1999 Grayson et al [9] / 5†† | 66 (mean) | 8 of 9 cases were Africans | NI | NI | NI | 4 of 9 cases had CIN I-III | 1 ASC, Clear cell adenocarcinoma; 1 BSCC; 1 ACC; 1 ASC; 1 combined ABC-ACC‖, BSCC, SCC | NI | NI | NI | NI | NI | NI |
| 14 | 2001 Grayson et al [10] / 2 | 68 | Asians | NI | NI | NI | CIN III | CS (ABC, BSCC, SCC, ACC as carcinomatous component) | TH (after cervical biopsy) | NI | NI | NI | NI | vaginal recurrence, NI |
| | | 55 | Africans | NI | NI | NI | CIN III | CS (ABC, BSCC, ACC as carcinomatous component) | cervical biopsy | NI | NI | NI | NI | NI |
| 16 | 2002 Takeshima et al [11] / 1 | 84 | Asians | pelvic mass | NA | cervical mass | CIN III | CS (SCC, adenocarcinoma as carcinomatous component) | TH (after cervical biopsy) | NI | TH, BSO, CT, RT | NA | abdominal wall (recurrent stage) | DOD, 4 |
| 17 | 2005 Parwani et al [12] / 9 | 70 | NI | abnormal Pap smear | ASCUS, AGUS | none | HSIL | Adenoid basal/squamous carcinoma | LEEP(with positive margin) | IB | RH, LND | negative | none | NED, 27 |
| | | 71 | NI | abnormal Pap smear | abnormal smear, not specified | none | HSIL | Adenoid basal/squamous carcinoma | Cone biopsy(with positive margin) | IB | TH, BSO, RT | NA | none | NED, 72 |
| | | 79 | NI | abnormal Pap smear | HSIL | none | HSIL | Adenoid basal/squamous carcinoma, SCNC | Cone biopsy(with positive margin) | IB | RT | NA | none | DOC, 18 |
| | | 81 | NI | abnormal Pap smear | abnormal smear, not specified | none | LSIL | SCC, ACC | Cone biopsy(with positive margin) | IB | RH, BSO, LND | negative | none | NED, 18 |
| | | 61 | NI | abnormal Pap smear | HSIL | cervical lesion, not specified | HSIL | Adenoid basal/squamous carcinoma | cervical biopsy | IB | VTH, RSO | NA | none | NED, 29 |
| | | 45 | NI | abnormal Pap smear | HSIL | cervical lesion, not specified | HSIL | Adenoid basal/squamous carcinoma | cervical biopsy | IB | TH, BSO, LND, RT | negative | none | NED, 84 |
| | | 64 | NI | pelvic relaxation | NA | none | HSIL | SCC | TH | IB | TH | NA | none | NED, 8 |
| | | 49 | NI | abnormal Pap smear | HSIL | none | HSIL | Adenoid basal/squamous carcinoma | LEEP(with positive margin) | IB | RH, BSO, LND | negative | none | recent follow-up |
| | | 65 | NI | uterine prolapse | NA | none | HSIL | Adenoid basal/squamous carcinoma | VTH | IB | VTH | NA | none | recent follow-up |
| 26 | 2005 Teramoto et al [13] / 1‡‡ | 59 | Asians | asymptomatic, abnormal Pap smear | abnormal smear, not specified | cervical lesion, not specified | none | SCC | cold-knife conization (after cervical biopsy) | IB1 | TH, LND | negative | none | NED, 18 |
| 27 | Current report / 1 | 64 | Asians | asymptomatic, abnormal Pap smear | scc | none | CIN III | SCC | RH | IB1 | RH,BSO,pelvic& para-aortic LND, CCRT | negative | none | NED, 6 |

**Abbreviations: ACC,adenoid cystic carcinoma; AGUS,atypical glandular cells of undetermined significance; ASC,adenosquamous carcinoma; ASCUS,atypical squamous cells of undetermined significance; BSCC,basaloid squamous cell carcinoma; BSO,bilateral salpingo-oophorectomy; CCRT,concurrent chemoradiotherapy; CIN,cervical intraepithelial neoplasia; CIS,carcinoma in situ; CS,carcinosarcoma; CT,chemotherapy; DOC,death from other causes; DOD,dead of disease; D&C,dilatation and curettage; HSIL,high grade squamous intraepithelial lesion; LEEP,loop electrosurgical excision procedure; LND,lymph node dissection; LSIL,low grade squamous intraepithelial lesion; NI,information not itemized in original manuscript; NA,not applicable; NED,no evidence of disease; PMB,postmenopausal bleeding; RH,radical hysterectomy; RSO,right salpingooophorectomy; RT,radiation therapy; TH,total hysterectomy; SCC,invasive squamous cell carcinoma; SCNC,small cell neuroendocrine carcinoma; VTH,total vaginal hysterectomy.** † Excluded the previous reported cases of adenoid basal carcinoma associated with microinvasive (FIGO stage IA1) squamous cell carcinoma[5,12]. ‡ Applied available information with 2009 FIGO staging for carcinoma of the cervix. § Data from one case in the ACC group of Ferry & Scully (1988), mean age was of patients in the ACC group. ‖ Equivalent proportion in admixed ABC and ACC. ¶ Included case which previously reported as ABC associated with microinvasive SCC in Brainard & Hart (1998). †† Data from 5 cases in total 9 cases of ABCs in Grayson et al (1999). ‡‡ 7 cases of ASC with an ABC-like lesion in Teramoto et al (2005) were excluded due to indefinite diagnosis of ABC.
